# Analyzing the vast coronavirus literature with CoronaCentral

**DOI:** 10.1073/pnas.2100766118

**Published:** 2021-05-20

**Authors:** Jake Lever, Russ B. Altman

**Affiliations:** ^a^Department of Bioengineering, Stanford University, Stanford, CA 94305

**Keywords:** coronavirus, literature categorization, machine learning, literature analysis

## Abstract

The SARS-CoV-2 pandemic has caused a surge in research exploring all aspects of the virus and its effects on human health. The overwhelming publication rate means that researchers are unable to keep abreast of the literature. To ameliorate this, we present the CoronaCentral resource that uses machine learning to process the research literature on SARS-CoV-2 together with SARS-CoV and MERS-CoV. We categorize the literature into useful topics and article types and enable analysis of the contents, pace, and emphasis of research during the crisis with integration of Altmetric data. These topics include therapeutics, disease forecasting, as well as growing areas such as “long COVID” and studies of inequality. This resource, available at https://coronacentral.ai, is updated daily.

The COVID-19 pandemic has led to the greatest surge in biomedical research on a single topic in documented history (Fig. 1*A*). This research is valuable both to current and future researchers as they examine the long-term effects of the virus on different aspects of society. Unfortunately, the vast scale of the literature makes it challenging to navigate. Machine-learning systems that can automatically identify topics and article types of papers would greatly benefit researchers who are searching for relevant coronavirus research.

**Fig. 1.**
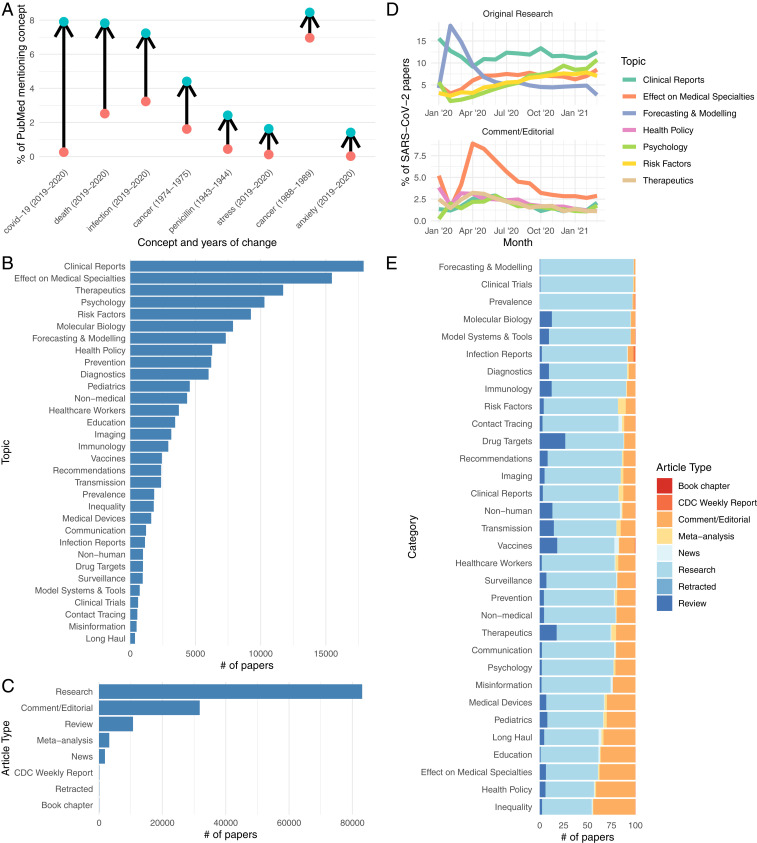
Overview of research trends and important topics. (*A*) Largest year-on-year changes in the percentage of papers that mention a biomedical concept using data from PubTator (8). (*B*) Frequency of each topic and (*C*) article type across the entire coronavirus literature. (*D*) The trajectories of the top five topics for original research and comment/editorial articles for SARS-CoV-2. (*E*) Different proportions of article types for each topic.

Analysis of the coronavirus literature was spurred by the availability of the CORD-19 literature dataset (1) and access to PubMed. Many approaches have used topic modeling techniques to extracted unsupervised topics of discussions (2, 3). The TREC-COVID–shared task provided several information retrieval challenges on specific COVID-19 topics (4). Other research implements advanced-search functionality to provide keyword search (5, 6). LitCovid provides a limited set of categories to index all literature (7).

Our approach improves on the existing methods, including LitCovid, by covering a larger set of papers with the inclusion of PubMed and CORD-19 along with SARS/MERS papers, a larger and more specific set of topics, identification of article types (e.g., Reviews), integration of Altmetric esteem data, and indexing by a wide set of biomedical terms (e.g., drugs, viral lineages, and so forth). All data are available for download and the full codebase is available on GitHub.

## Results

To provide more detailed and higher-quality topics, we pursue a supervised learning approach and have annotated over 3,200 articles with a set of 32 topics and 8 article types (Fig. 1 *B* and *C*). Individual papers may be tagged with multiple topics and typically a single article type. Using a BERT-based document multilabel classification method, we achieved a micro-F1 score of 0.68 with microprecision of 0.76 and microrecall of 0.62. A breakdown of the performance by topics and article types shows varying quality of performance, with some performing very well (e.g., contact tracing and forecasting) and others performing poorly (e.g., long haul), likely due to extremely low representation in the test set. Several other topics and article types are identified using simple rule-based methods, including clinical trials and retractions.

As of 3 March 2021, CoronaCentral covers 128,921 papers. The top topic (Fig. 1*B*), Clinical Reports, covers articles describing patients and their symptoms, including case reports. The second top topic, the Effect on Medical Specialties, covers how specific specialties (e.g., oncology) must adapt to the pandemic. While other approaches have focused on viral biology, we made a specific effort to also identify papers that discuss societal impacts, including the psychological aspects, the inequality highlighted by the pandemic, and the long-term effects of COVID. This final topic, also known as “long COVID,” is covered by the Long Haul topic, which currently includes 362 papers. We find the first Long Haul COVID papers appeared in April 2020, and there has been a slow steady increase in publications since then, with ∼30 papers per month recently. While all of the annotated Long Haul documents used to train our system focus on SARS-CoV-2, our system finds 12 papers for the long-term consequences of SARS-CoV and one for MERS-CoV. Our approach also identifies the article type, which is important, given our estimate that 24.7% of publications are comments or editorials and not original research (Fig. 1*C*).

**Fig. 2.**
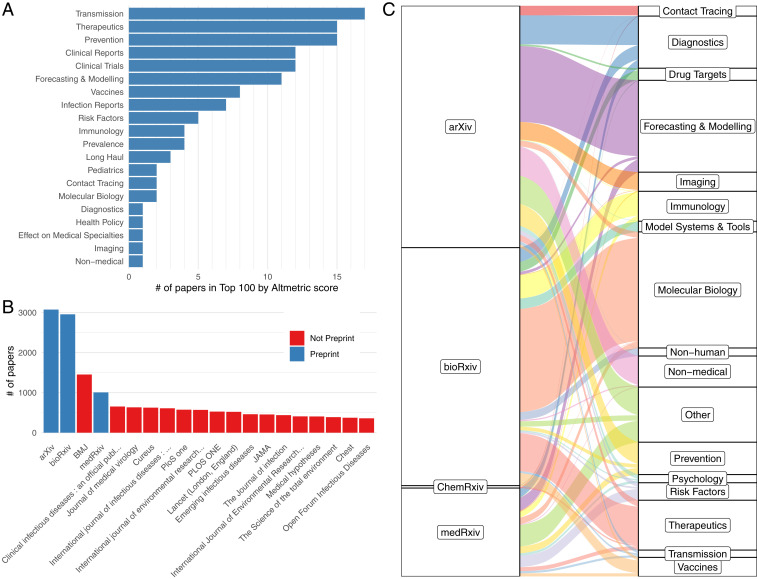
Communication of research has changed with a greater emphasis on social media and preprint servers. (*A*) The number of papers categorized with each topic in the 100 papers with highest Altmetric scores. (*B*) Top journals and preprint servers. (*C*) Topic breakdown for each preprint server and nonpreprint peer-reviewed journals. Infrequent topics in preprints are grouped in “Other.”

The predicted topics reveal the publication trend during the pandemic (Fig. 1*D*). Early research focused on disease forecasting and modeling, which has steadily decreased as a proportion as other opics, such as the risk factors of coronavirus, have increased. Clinical reports have been steady, as a proportion, throughout the pandemic. In commentaries and editorials, the main topic has been the effect on different medical specialties. Fig. 1*E* shows that different topics have drastically different distributions of article types. While almost all papers that look at forecasting or modeling are original research, about half of the health policy articles are commentary or editorials. Notable topics with larger proportions of reviews are the more science-focused topics, including molecular biology, drug targets, and vaccines. To identify highly discussed papers and make the resource more navigable, we integrated Altmetric data to identify papers that have received wide coverage in mass and social media. Fig. 2*A* shows the breakdown of topics in the 100 papers with highest Altmetric scores. The distribution contrasts with the overall distribution of coronavirus literature, reflecting the interest in treatments and prevention methods.

## Discussion

Preprint servers have played an important role in disseminating research during this crisis (Fig. 2*B*). However, they only account for 5.8% (7,011 of 121,419) of all SARS-CoV-2 articles. We find that the indexed preprint servers were used for dramatically different topics (Fig. 2*C*). As might be expected, the more mathematically focused papers, such as forecasting/modeling, have been submitted to *arXiv*. Molecular biology tends to go to *bioRxiv*, therapeutics to *ChemRxiv*, and a diverse set of clinical topics to *MedRxiv*.

The pandemic has revealed many challenges of communicating important research during a health crisis. Pre-Covid methods for literature search often relied on long-term metrics, like citation counts, to prioritize search results. These approaches are unsuitable in a fast-moving environment. By integrating Altmetric scores with detailed topic and article-type information, CoronaCentral (https://coronacentral.ai) enables users to narrow their focus to identify important papers in a timely manner. As the pandemic continues, monitoring of the trending articles will help identify new topics and verify that topic drift does not noticeably reduce machine-learning quality.

## Materials and Methods

The documents from PubMed and CORD-19 are processed with a pipeline for topic and article type prediction, data cleaning, and other steps, described in *SI Appendix*, *Extended Methods*. Detailed information is available at the GitHub repository (https://github.com/jakelever/corona-ml), with data at https://doi.org/10.5281/zenodo.4383289.

## Supplementary Material

Supplementary File

## Data Availability

The coronavirus articles with topics/article types and extracted entities data have been deposited in Zenodo (DOI 10.5281/zenodo.4383289).
