# Supplementary material for: Analyzing the vast coronavirus literature with CoronaCentral
Source: Proc Natl Acad Sci U S A. 2021 May 20;118(23):e2100766118. doi: 10.1073/pnas.2100766118 (PMC8202008; doi:10.1073/pnas.2100766118)
Supplement: Supplementary File [file pnas.2100766118.sapp.pdf]

Extended Methods for “Analyzing the vast coronavirus literature with CoronaCentral”

Data Collection: The CORD-19 dataset (1) and PubMed articles containing relevant coronavirus keywords are downloaded daily. Articles are cleaned to fix Unicode issues, remove erroneous text from abstracts, and identify publication dates. Non-English language articles are filtered out using a rule-based system based on sets of stopwords in multiple languages. To remove duplicates, documents were merged using identifiers, combinations of title and journal, and other metadata. Metadata from the publishers’ websites is also integrated which enables normalization of consistent journal names and further abstract text fixes. Additional manual fixes to title, abstracts, and metadata are applied to the corpus. Altmetric data is updated regularly and integrated with the data.

Topics and Article Types: Manual evaluation of an initial 1000 randomly selected articles was undertaken to produce a draft list of topics (e.g. Therapeutics) and article types (e.g. Comment/Editorial). An iterative process was undertaken to adjust the topic and article type list to provide better coverage for the curated documents. A further 500 documents were sampled later in the pandemic and another iterative process was undertaken as new topics were appearing in larger quantities (e.g. contact tracing). Finally, manual review of the papers with high Altmetric scores identified several smaller topics that had not been captured by random sampling, including Long Haul, which were added to the list. As the coronavirus literature grows, we may need to add new topics as the research focus changes.

Annotation: Articles were manually annotated for topics and article types using a custom web interface. The Research article type was omitted, with the assumption that any article type without article type annotation was an Original Research article. The first 1500 randomly sampled articles were annotated during the iterative process that defined the set of topics and article types. This first set illustrated temporal skewing of topics (outlined in Fig 1D) as papers sampled earlier in the pandemic tended to include more Forecasting papers. A further ~1200 articles have been identified for annotation through manual identification, their high Altmetric scores or uncertainty in the machine learning system. Some of the articles were flagged using the CoronaCentral “Flag Mistake” system while others were identified through manual searching to improve representation of different topics. A final 500 articles were randomly selected and annotated for use as a held-out test set.

BERT-based Topic & Article Type Prediction: Cross-validation using a 75%/25% training/validation split was used to evaluate BERT-based document classifier as well as traditional methods. Topics and article types were predicted together using the title and abstract as input. Multi-label classifiers were implemented using ktrain (2) and HuggingFace models for BERT models and scikit-learn for others (3). Hyperparameter optimization involved a grid search over different models (BioBERT, BlueBERT, PubMedBert and scibert), epochs (4 to 96) and learning rate (1e-3 to 5e-6) and selecting for the highest macro F1 score. The best model used the microsoft/BiomedNLP-PubMedBERT-base-uncased-abstract BERT model (4) with 32 epochs, a learning rate of 5e-05, and a batch size of 8. More details of the hyperparameter optimization are available in the GitHub repository. This model was then evaluated on the held-out test set for final performance and a full model was retrained using these parameters with all annotated documents and applied to the full coronavirus literature. To match with the annotated data where a document without a specific article type is assumed to be Original Research, any

document that has not been assigned an article type by the BERT classifier is predicted as an Original Research article.

Additional Rule-based Topic & Article Type Prediction: Additional heuristics were used to identify the Clinical Trial topic (which is not predicted by the BERT system) and to overrule the article type prediction made by BERT when additional information was available. Clinical trials were identified through regular expression search for trial identifiers (which through validation on 100 randomly selected papers tagged as Clinical Trial, showed 93% accuracy for papers discussing trial results or protocols). For article types: book chapters were identified by obvious chapter headings in document titles, CDC Weekly Reports by the specific journal name ('MMWR. Morbidity and Mortality Weekly Report') and retractions through PubMed flags and titles beginning with "Retraction", "Retracted" or "Withdrawn". The metadata provided by the publisher's website is combined with PubMed metadata to identify some article types, e.g. documents tagged as Commentary or Viewpoints on publisher's websites were categorized as Comment/Editorial.

Entity Extraction: In order to enable users to search by a specific biomedical entity (e.g. a drug name) and for the search to capture all relevant synonyms, we extract mentions of biomedical concepts and map them back to their normalized forms with unique identifiers. This set of entity types include drug names, locations, genes/proteins, symptoms and other types. This set was refined based on entities that would be particularly relevant for different topics (e.g. Drug for Therapeutics, Symptom for Clinical Reports, etc). The lists of entities were sourced from WikiData or built manually. Entities of types Drug, Location, Symptom, Medical Specialty, and Gene/Protein are gathered from Wikidata using a series of SPARQL queries. A custom list of Prevention Methods, Risk Factors, Test Types, Transmission Types, and Vaccine Types is also constructed based on Wikidata entities. Additional customizations are made to remove incorrect synonyms. A custom list of coronavirus proteins was added to the Gene/Protein list. Exact string matching is used to identify mentions of entities in text using the Wikidata set of synonyms and a custom set of stopwords. A simple disambiguation method was used to link virus proteins with the relevant virus based on mentions of the virus elsewhere in the document. This meant that a mention of a "Spike protein" in a MERS paper would correctly link it to the MERS-CoV spike protein and not to the SARS-CoV-2 spike protein. If multiple viruses were mentioned, no disambiguation was made. A regular expression based system is used to identify mentions of Genomic Variation (e.g. D614G) and viral lineages (e.g. B.1.1.7)

Interface: The data is presented through a website built using NextJS with a MySQL database backend. Visualizations are generated using ChartJS and mapping using Leaflet.

PubTator Concept Analysis: To find the concepts that have had the largest difference in frequency, PubTator Central (5) was used as it covers a broad range of biomedical entity types such as disease, drug, and gene. It was aligned with PubMed to link publication dates to entity annotations. This used the BioText project (https://github.com/jakelever/biotext). Concept counts were calculated per publication year and normalized to percentages by total publications by year. The differences between these ordered to identify the biomedical concepts with largest change in percentage. Entity mentions of the type "Species" were removed due to lack of value as "human" dominated the data.

Other Analyses: All other analyses were implemented in Python and visualized using R and ggplot2.

Code Availability: The code for the machine learning system and paper analysis are available at https://github.com/jakelever/corona-ml. The code for the web interface is available at https://github.com/jakelever/corona-web.

Data Availability: The data is hosted on Zenodo and available at https://doi.org/10.5281/zenodo.4383289.

References for Extended Methods

1. L. L. Wang et al., CORD-19: The COVID-19 Open Research Dataset in Proceedings of the 1st Workshop on NLP for COVID-19 at ACL 2020, (Association for Computational Linguistics, 2020).
2. S. Maiya, ktrain: A low-code library for augmented machine learning. arXiv arXiv:2004.10703 [cs.LG] (2020).
3. F. Pedregosa et al., Scikit-learn: Machine learning in Python. Journal of Machine Learning Research 12, 2825–2830 (2011).
4. Y. Gu et al., Domain-Specific Language Model Pretraining for BiomedicalNatural Language Processing. arXiv preprint arXiv:2007.15779 (2020).
5. C.-H. Wei, A. Allot, R. Leaman, Z. Lu, PubTator central: automated concept annotation for biomedical full text articles. Nucleic acids research 47, W587–W593 (2019).
